# Examination of language, cognitive, and mathematical skills in childhood endocrine diseases

**DOI:** 10.3389/fpsyg.2024.1284950

**Published:** 2024-10-03

**Authors:** Ebrar Çavuşoğlu, Merve Savaş, Emine Dilek, Yusuf Elgörmüş, Senanur Kahraman Beğen

**Affiliations:** ^1^Department of Speech and Language Therapy, Faculty of Health Sciences, Istanbul Atlas University, Istanbul, Türkiye; ^2^Faculty of Medicine, Istanbul Atlas University, Istanbul, Türkiye

**Keywords:** childhood endocrinologic disease, language, cognition, mathematical performance, child

## Abstract

**Introduction:**

Children diagnosed with endocrine disorders may exhibit atypical development and may encounter challenges in language, academic, and cognitive skills, as well as social–emotional issues. The objective of this study was to identify potential therapeutic requirements in the areas of language, cognition, and mathematical skills among children with endocrine disorders who experience school failure. This will enable an early evaluation of speech and language disorders and the planning of interventions to be possible.

**Methods:**

In this study, children with endocrine disorders were compared with their normally developing peers in terms of language, cognition, mathematical skills, and psychosocial characteristics. In this study, 15 children diagnosed with endocrine disorders (8 females, 7 males; mean age: 10, SD: 2) and 15 children with normal development (8 females, 7 males; mean age: 10, SD: 2) participated. The participants were subjected to the Test of Language Development-Primary: Fourth Edition Turkish Revision (TOLDP-4:T), the Turkish Nonword Repetition Test (TNRT), the Turkish Multilingual Sentence Repetition Test (LITMUS-TR), the Wechsler Intelligence Scale for Children (WISC-R), the Problem-Solving Test (PST), the Revised Child Anxiety and Depression Scale-Child Version (RCADS-CV), the Coopersmith Self-Esteem Inventory (CSEI), and the Child Behavior Checklist (CBCL).

**Results:**

The findings of the study indicate that children with endocrine disease have lower performance in language, cognition, and mathematical skills compared to their healthy peers. Otherwise, they do not differ in terms of social–emotional status assessed by psychological scales.

**Discussion:**

These findings suggest that while children with endocrine disorders face challenges in academic and cognitive domains, their social-emotional development remains relatively unaffected. Early identification and intervention in language, cognition, and mathematical skills may help address the academic struggles of these children, potentially improving their school performance and overall well-being.

## Introduction

Childhood chronic diseases are medical conditions that can be diagnosed using reproducible and valid methods. According to scientific standards, in children aged 0–18 years, for whom there are no definitive treatment protocols yet, these conditions persist for more than 3 months or potentially last longer than 3 months and recur three or more times in the last year (Mokkink et al., 2008). These diseases may restrict daily life as they necessitate regular medical attention, follow-up, care, or hospitalization (Miodovnik et al., 2011). They may also interfere with children’s language, cognition, and social development (Jones et al., 2017) and result in school failure (Forrest et al., 2011).

In endocrine diseases, which are regarded as childhood chronic diseases, developmental delays, social–emotional problems, academic skill difficulties, and school failure can be observed due to medical conditions directly associated with the disease and/or psychosocial effects caused by the disease (Ahmed et al., 2021; Salerno et al., 2020). Mathematics performance, which is considered one of the important indicators of school success (Watts et al., 2014), is critical for both personal and professional success and is closely linked to life satisfaction, health, income, and employability (Lipnevich et al., 2016).

The few studies examining the academic skills of children with endocrinological diseases suggest that disease-specific factors negatively affect mathematical skills (Garcy, 2009). On the other hand, diabetes, an endocrine disease, is not directly related to mathematics performance (Crump et al., 2013). The contradictory results in the literature suggest that there is a need for further research into learning disabilities and mathematical underachievement caused by certain endocrinological diseases.

### Language and cognitive disorders in various childhood Endocrinologic disorders

#### Language, cognitive, and academic problems in precocious puberty

Precocious puberty is defined as the onset of secondary sexual characteristics prior to the age of 8 years in girls and before the age of 9 years in boys and is attributed to the early maturation of the hypothalamic–pituitary-gonadal axis. Despite the early chronological onset, the pattern and sequence of pubertal events are generally considered normal (Taş and Haspolat, 2019).

When the language functions of subjects exposed to elevated sex hormone levels were examined, significant differences were found between children exposed to high androgen and estrogen. Although the estrogen-exposed group achieved higher scores in semantic-dependent tasks, the androgen-exposed group achieved higher scores in automatic syntactic functions and memory-dependent tasks (McCardle and Wilson, 1990). It has been revealed that the frequency of depression in precocious puberty cases is higher than that of peers with normal development (Huang et al., 2021).

#### Language, cognitive, and academic problems in obesity

Obesity is defined as the accumulation of excess body fat. The body mass index, which is the ratio of weight to the square of height, is used to define obesity clinically (Babaoğlu and Hatun, 2002). Obesity is caused by many monogenetic factors. Obesity may be associated with various genetic syndromes, resulting in phenotypes such as dysmorphic or developmental delay (Kostovski et al., 2017). The greatest risk factor for obesity is genetic inheritance. Obesity in children is correlated with parental obesity. Obesity in both parents contributes to the rate of obesity in their children (Garn et al., 1976). When behavioral risk factors are considered, it is found that sleep patterns, lifestyle, and stress affect obesity (Brown et al., 2015).

When the emotional and social aspects of childhood obesity are evaluated, the person’s daily quality of life decreases due to obesity. Obesity has many psychosocial complications, such as depression, body dissatisfaction, stigmatization, and low self-esteem (Vander Wal and Mitchell, 2011). It has been revealed that there is a significant relationship between being overweight and having lifelong major depressive illnesses in children older than 8 years of age (Gibson-Smith et al., 2020). Obesity can affect school attendance with its negative effects on physical and mental health (An et al., 2017). Obesity decreases reading and mathematics performance and may impair self-regulation skills by causing expression and internalization problems (Judge and Jahns, 2007). It is also emphasized that obesity may impair learning and memory performances in children and adolescents due to physical problems such as sleep apnea (Pyle and Poston, 2006).

#### Language, cognitive, and academic problems in Type 1 diabetes

Diabetes is a chronic, metabolic, and autoimmune disease that is characterized by hyperglycemia (Sağlam, 2004). Type 1 diabetes is one of the common pathologies in children (Lowes et al., 2015) and its prevalence is gradually increasing without any known cause (Pilapil et al., 2016; Burner et al., 2014). A child diagnosed with type 1 diabetes is 2.3 times more likely to experience mental health problems than other children (Cooper et al., 2017). During puberty, when rapid changes occur both physiologically and psychosocially, young people with diabetes are prone to higher rates of stress and psychological illness (Boogerd et al., 2014; Stahl-Pehe et al., 2017). Treatment options include pharmacological drugs, self-control, and lifestyle changes (Fortin et al., 2017). Changes in life habits and style can lead to psychosocial problems such as depression or eating disorders (Abubakari et al., 2016). Therefore, the comprehensive management of type 1 diabetes necessitates a multidisciplinary approach.

Individuals with prediabetes or diabetes are more at risk for cognitive impairment (Luchsinger et al., 2018). In a study examining the cognitive skills of children and adolescents with type 1 diabetes, it was observed that the rate of cognitive impairment increased with the decrease in age of diagnosis (Ryan, 2006).

#### Language, cognitive, and academic problems in thyroid disease

Thyroid hormone levels within normal ranges are essential for neurocognitive development and metabolism, as well as for physical growth during the growing age (Niedziela, 2021). Thyroid pathologies that result in hyperthyroidism during childhood, such as congenital or acquired hypothyroidism and Graves’ disease, can lead to persistent cognitive decline and school failure (Perri et al., 2021). Subclinical hypothyroidism is associated with improved performance in certain areas of cognitive functioning, while subclinical hyperthyroidism may be a potential risk factor (Wu et al., 2006).

### The relationship between language and mathematical skills

Language is considered to be a code system that enables communication between people. It overlaps with mathematics in that it contains spoken or written symbols that represent ideas or images and has an abstract and consistent system of rules (Wakefield, 2000). Language proficiency is an essential skill for academic success. Preschool children who possess advanced language skills exhibit higher levels of school readiness and overall math performance. However, many students with math difficulties are more likely to experience difficulties with language skills than their peers. Research into the relationship between various components of language and mathematics achievement shows that morphosyntactic skills have a direct impact on mathematics performance (Chow et al., 2021) and that semantic and pragmatic skills contribute to various mathematical learning domains such as geometry and fractions (Kleemans and Segers, 2020). Similarly, phonological skills have been found to predict arithmetic, as the solution to arithmetic problems is based on verbal codes of a phonological nature (Simmons and Singleton, 2008). Since both have a common syntax, morphosyntactic skills have an impact on arithmetic performance. In other words, the syntax of a sentence and the order of items in arithmetic operations have a computational load on working memory (Baldo and Dronkers, 2007).

A mathematical assessment is a detailed presentation of the child’s strengths and weaknesses along with an age-appropriate curriculum. In this context, descriptive approaches should be used in addition to standard assessments to reveal mathematical skills and areas that require further support (Chiang and Lin, 2007). One of the descriptive approaches used in mathematical assessment is the observation of the whole process from beginning to end during the solution of verbal problems (Maharani et al., 2019). Verbal problems are divided into routine and non-routine problems. Routine problems are mathematical expressions that adhere to a simple, linear syntax and are relatively brief, typically consisting of approximately three main sentences. Non-routine problems, however, are structures in which syntactic information is presented, information that may be redundant or superficial, and a context that is functional for problem solving. For this reason, non-routine problems are those that require more thought than routine problems and where the solution to the issue is not obvious. The context presented in the problem is functional and critical for the solution (Beghetto, 2017; Strohmaier, 2020). Linguistic, arithmetic, spatial, and general reasoning skills have an impact on solving verbal problems. However, linguistic skills are the most consistent predictors of solving non-routine problems. Arithmetic skills predict correct solutions to problems involving only computation, spatial skills predict solution rates in the presence of a visual representation, and general reasoning skills are more closely related to simpler problems that can be easily solved (Strohmaier et al., 2022).

It is suggested that language structure and individual differences in language development are closely related to mathematical performance. Based on experimental studies testing the effects of supporting language development on mathematical achievement, it is concluded that increasing linguistic skills significantly improves performance in mathematical domains (Espinas and Fuchs, 2022). Given the determinant effect of language development level on mathematical performance, it is necessary to evaluate mathematical skills and language development together.

This study aimed to identify possible therapeutic needs in the areas of language, cognition, and mathematical skills of children with endocrine disorders who experience school failure.

## Materials and methods

### Participants

15 children who were followed up in the pediatric endocrinology outpatient clinic and whose families reported school failure were included in the study. The study included participants with any of the diagnoses of precocious puberty, obesity, type 1 diabetes mellitus, or thyroid disease (collectively known as endocrine disorders [ED]) who had been followed up in a pediatric endocrinology outpatient clinic for at least 6 months. The control group consisted of 15 age-matched, healthy controls (HC). The inclusion criteria for sample selection were as follows: having any of the diagnoses of precocious puberty, exogenous (simple) obesity, type 1 diabetes mellitus, developmental delay, or thyroid disease by a pediatric endocrinologist, being at least 6 years old, not having a diagnosis of autism spectrum disorder, and being consented to participate in the study by the family. The exclusion criteria for the sample selection were as follows: absence of precocious puberty, obesity, type 1 diabetes or thyroid disease, developmental delay by a pediatric endocrinologist, being younger than 6 years of age, having a diagnosis of autism spectrum disorder, not having been given consent by the family to participate in the study, and presence of an additional disease or a concomitant syndromic condition. The inclusion criteria for the control group included the absence of any primary neurologic or psychiatric disease diagnosis and the absence of any childhood chronic disease diagnosis.

As a result of the meeting held on 15 June 2022, approval was obtained from the Istanbul Atlas University Non-Interventional Scientific Research Ethics Committee, with the decision numbered E-22686390-050.99-19958. This study was conducted at the Istanbul Atlas University Hospital and Clinic.

Children with a history of school failure reported by their families were selected for inclusion in the study. A series of tests were used to assess various psychological and cognitive characteristics of the children, including language development, cognitive skills, problem-solving skills, anxiety and depression levels, self-esteem, and behavior. To assess language development, the Test of Language Development-Primary: Fourth Edition Turkish Revision (TOLDP-4) was used to make a differential diagnosis of language disorders in children aged 4–8 years and to measure the dimensions of language development. The Turkish Nonword Repetition Test (TNRT) was used for the early diagnosis of specific language disorders. The Turkish Multilingual Sentence Repetition Test (LITMUS-TR) was used to assess children’s morphosyntactic abilities. The Wechsler Intelligence Scale for Children (WISC-R) was utilized to assess the cognitive abilities of the children. The Problem-Solving Test (PST), which includes various strategies, was administered to measure problem-solving skills. The Revised Child Anxiety and Depression Scale-Child Version (RCADS-CV) was used to assess anxiety and depression levels. The Coopersmith Self-Esteem Inventory (CSEI) was used to measure self-esteem. The Child Behavior Checklist (CBCL) was used to assess children’s behavior and social adjustment. These tests were selected to provide a comprehensive evaluation of the children’s psychological and cognitive status.

### Data collection tools

#### Test of Language Development-Primary: Fourth Edition Turkish Revision (TOLDP-4:T)

The TOLDP-4:T, which was adapted into Turkish and subjected to validity and reliability studies by Topbaş and Güven (2017), is a norm-dependent standardized test used in children aged between 4 years and 8 years and 11 months for the differential diagnosis of language disorder and to measure the dimensions of language development. The TOLDP-4:T consists of nine subtests, of which the first six are fundamental and the subsequent three are supplementary. The basic subtest categories are Picture Vocabulary, Associated Vocabulary, Word Description, Sentence Comprehension, Sentence Repetition, and Morphological Completion. Supplementary subtests include Word Discrimination, Phonemic Analysis, and Articulation (Topbaş and Güven, 2017).

#### Turkish nonword repetition test (TNRT)

It was aimed at early diagnosing monolingual and bilingual children in terms of specific language disorders. The words in the test were generated based on the phonotactic and orthographic structure of Turkish, as well as the structure and frequency of syllables in Turkish. Another criterion in the creation of the nonwords in the test was their resemblance to meaningful words in Turkish. At the end of some language-like words, a construction or inflectional suffix consisting of a consonant-vowel-consonant combination was added to examine the contribution of Turkish construction and inflectional suffixes to the success of nonword repetition (Topbaş et al., 2014).

#### The Turkish Multilingual Sentence Repetition Test (LITMUS-TR)

It was aimed at early diagnosis of morphosyntactic abilities of monolingual and bilingual school-age children who speak Turkish. In this test consisting of 30 sentences, the child is required to repeat the sentence correctly immediately after listening to it. During the development of the test, it was ensured that the sentences were appropriate to the developmental level of the children, did not contain figurative expressions, and consisted of at least 4 and at most 11 words. The test items were comprised of five different categories, namely subject-object-predicate sequence, what-who questions, noun phrases, relative clauses, clausal attachments, and conditional structures. The child’s productions are documented on the administration form of the test and are audio-recorded. There exist various scoring formats for the responses provided to the test items, including scoring according to the number of correct repetitions, scoring according to the number of repetition errors, scoring according to syntactic structure, and scoring according to lexical errors. According to the true-false scoring format, if the child produces the entire sentence correctly, it is scored as 1; if it is incorrect, it is scored as 0 (Topbaş, 2021).

#### Wechsler Intelligence Scale for Children (WISC-R)

It is the 1974 revised version of the Wechsler Intelligence Scale for Children (WISC), developed in 1949 by Wechsler. It is a clinical measurement tool that measures the cognitive abilities of children applied individually. The WISC-R consists of two sections: verbal and performance. There are 6 subtests in each section. According to the study, the verbal subtests included General Knowledge, Similarities, Arithmetic, Comprehension, and Number Sequences, while the performance subtests comprised Picture Completion, Picture Arrangement, Block Design, Object Assembly, and Coding (Savaşır and Şahin, 1995).

#### Problem-Solving Test (PST)

It is a test consisting of a total of 18 questions, including the 9 most commonly used problem-solving strategies in primary and secondary schools, with two open-ended questions from each strategy. Common problem-solving strategies are “Draw a Diagram,” “Look for a Pattern,” “Simplify the Problem,” “Make a Table,” “Make a Systematic List,” “Use a Variable,” “Logical Reasoning” and “Working Backwards.” The classification of strategies was determined based on the formulation, execution, interpretation, and evaluation processes used in solving the problem (Temel and Altun, 2020).

#### The Revised Child Anxiety and Depression Scale-Child Version (RCADS-CV)

Developed by Chorpita et al. based on DSM-IV diagnostic criteria to assess depression and anxiety in children and adolescents. The RCADS consists of 47 items. In the 4-point Likert-type scale (never = 0, sometimes = 1, often = 2, and always = 3), parents are asked to fill in the items, including the frequency of anxiety and depression-related symptoms and behaviors in their child. In addition to total anxiety (sum of five anxiety subscales) and total anxiety-depression (sum of all subscales), it includes six subscales: separation anxiety, general anxiety, panic, social phobia, obsession/compulsion, and depression. Turkish adaptation, validity, and reliability studies were conducted by Gormez et al. (2017).

#### Coopersmith Self-Esteem Inventory (CSEI)

It was developed by Stanley Coopersmith in 1967 and can be applied to all ages. The concept of self-esteem in the scale, which is used to evaluate one’s attitudes toward oneself in various areas, is also associated with some of the characteristics that one approves or disapproves of oneself. The self-esteem definition used in this scale has three features. Self-esteem is a judgment that reflects a general evaluation of oneself. This judgment possesses relative continuity and does not change immediately. This judgment may differ depending on the person’s age, gender, and various positions depending on social roles. The Coopersmith Self-Esteem Scale is a paper-and-pencil test consisting of 25 items that can be marked on the questionnaire as “Suits me” or “Does not suit me.” These items include statements regarding the person’s outlook on life, family relationships, social relationships, and resilience. The points given to each answer vary from 1 to 4. Different answers, items are left blank, or items where both options are checked, receive 0 points. Scores range from 0 to 25 or 0 to 100. A high score is considered to have a high self-esteem. The Cronbach’s alpha value for the positive dimensions of the self-esteem assessment scale short form was found to be 0.875 for 10 items, while the Cronbach’s alpha value of the ten-item negative dimensions measuring the negative dimensions of the self was found to be 0.853. When Cronbach’s alpha value was examined without creating two halves of the BPSAS-SF, it was found to be 0.897 (Sezer, 2001).

#### Child Behavior Checklist (CBCL)

It was developed by Achenbach and Edelbrock (1983) to assess the competence areas and problematic behaviors of children and youth aged 4–18 years in accordance with the information obtained from their parents. The scale consists of 20 competences and 118 problem items. Competence includes sports and non-sports activities in which the child and youth are actively involved and the work they do at home or outside the home. The rating is based on the amount and quality of participation. It also determines their functioning within social spheres. These are functions such as membership in a sports or social organization, club or group, relationships with friends, siblings, and parents, playing games, or doing work on their own. This reflects the achievement status at school, problems, and the quality and quantity of participation in school activities. The total competence score is obtained from the sum of the activity, sociability, and school subscales. The internal consistency of the scale was calculated using Cronbach’s alpha coefficient over the scores of the study sample. The coefficients were found to be: Introversion = 0.82, Extraversion = 0.81, and Total Problem = 0.88 (Dumenci et al., 2004).

### Data analysis

The data of the study were obtained by filling in the forms of the evaluation tools. The scores of all of these forms were separately calculated and were determined according to the age of the participants. Verbal language score in TOLDP-4:T, the total score in LITMUS-TR, the total score in TNRT, the total score in WISC-R, the total score in CSEI, the total score in problem items in the CBCL, the total score in the RCADS-CV, and the total score in the PST. The PST strategies used by the participants and the processes they used while solving the strategies were examined and calculated separately for each participant by two separate speech and language therapists at separate times. The results were compared, reconciled, and recorded. Statistical Package for the Social Sciences (SPSS; version 25) was used for data analysis. Percentage, minimum and maximum values, mean, and standard deviation values were used in the analysis of descriptive data. Because the data were not normally distributed and there were two groups, the Mann–Whitney U test—a nonparametric test—was used. In the analyses, *p* < 0.05 was taken as the significance value.

## Results

In this section, the findings obtained through the analysis of the data collected from the participants and the explanations made based on these findings are included.

The demographic information about the participants in the study group is given in Table 1. The proportion of healthy participants was 53.3 and 46.7% for females and males, respectively. There were no significant differences between the groups according to gender (*p* > 0.05) and age (*p* > 0.05) (Table 1). The diagnosis, age at diagnosis and medication used by participants with endocrine disorder are shown in Table 2.

**Table 1:** Demographic information of the participants.

Group		HC		ED		Difference
		*N*	%	*N*	%	*p*
Gender	Female	8	53.3	8	53.3	1.000
	Male	7	46.7	7	46.7	
Age		X̄	SD	X̄	SD	*p*
		10	2	10	2	0.806

ED, Endocrine Disorder. HC, Healthy Control; Mann–Whitney U Test, p < 0.05*..

**Table 2:** Clinical parameters of children with endocrine disorder.

	Diagnosis	Age of diagnosis	Medication
ED 1	Precocious puberty	10	Leuprolide acetate
ED 2	Hypothyroidism	6	Levothyroxine
ED 3	Precocious puberty	8	Leuprolide acetate
ED 4	Obesity	9	_
ED 5	Obesity	10	_
ED 6	Type 1 diabetes	10	Insulin
ED 7	Obesity	11	_
ED 8	Type 1 diabetes	8	Insulin
ED 9	Hyperthyroidism	8	Propylthiouracil
ED 10	Precocious puberty	9	Leuprolide acetate
ED 11	Precocious puberty	8	Leuprolide acetate
ED 12	Hypothyroid	15	_
ED 13	Obesity	7	_
ED 14	Hypothyroidism	7	Levothyroxine
ED 15	Type 1 diabetes	11	Insulin

ED, Endocrine Disorder.

In Table 3, descriptive statistics are given about the measurement tools used by all participants in the study. Table 3 shows the Mann–Whitney U test results for the comparison of the mean rank of the measurement tools of the participants with normal and endocrine disease. Accordingly, normal participants exhibited TOLDP-4:T (*p* < 0.001), TNRT (*p* = 0.012), WISC-R (*p* = 0.000), WISC-R verbal IQ (*p* < 0.001), WISC-R information (*p* = 0.018), WISC-R comprehension (*p* < 0.001), WISC-R similarities (*p* = 0.029), WISC-R vocabulary (*p* = 0.027), WISC-R performance IQ (*p* < 0.001), WISC-R picture completion (*p* = 0.003), WISC-R picture arrangement (*p* = 0.046), PST (*p* < 0.007), PST strategy (*p* = 0.007), and PST process (*p* = 0.006) were significantly higher than the mean scores of participants with endocrine disease.

**Table 3:** Comparison of test results of participants.

	Group	*N*	Min	Max	X̄	SD	*p*
TOLDP-4:T	HC	15	107.00	128.00	118.13	5.97	0.000*
	ED	15	87.00	118.00	103.67	9.87	
TNRT	HC	15	10.00	16.00	14.00	1.81	0.012*
	ED	15	9.00	15.00	12.13	1.88	
LITMUS-TR	HC	15	20.00	30.00	26.73	2.40	0.127
	ED	15	16.00	30.00	23.93	5.01	
WISC-R	HC	15	88.00	124.00	113.60	9.64	0.000*
	ED	15	59.00	114.00	91.20	15.92	
WISC-R	HC	15	80.00	126.00	106.33	11.17	<0.001*
Verbal IQ	ED	15	44.00	107.00	86.60	18.46	
WISC-R	HC	15	3.00	14.00	9.73	2.69	0.018*
Information	ED	15	4.00	11.00	7.8	1.97	
WISC-R comprehension	HC	15	8.00	16.00	11.53	1.64	<0.001*
	ED	15	0.00	12.00	7.27	3.27	
WISC-R	HC	15	6.00	15.00	10.93	2.43	0.062
Arithmetic	ED	15	4.00	14.00	11.53	2.42	
WISC-R	HC	15	8.00	17.00	12.53	2.47	0.029*
Similarities	ED	15	0.00	14.00	9.53	4.12	
WISC-R	HC	15	3.00	14.00	9.73	2.99	0.025*
Vocabulary	ED	15	2.00	16.00	7.40	3.44	
WISC-R Performance IQ	HC	15	110.00	126.00	115.20	8.12	<0.001*
	ED	15	44.00	120.00	95.53	18.76	
WISC-R	HC	15	10.00	16.00	12.73	1.58	0.003*
Picture completion	ED	15	5.00	15.00	9.20	3.12	
WISC-R	HC	15	6.00	15.00	11.00	2.80	0.046*
Picture arrangement	ED	15	5.00	14.00	8.76	3.33	
WISC-R	HC	15	9.00	18.00	12.80	2.37	0.054
Block design	ED	15	4.00	15.00	10.67	2.92	
WISC-R	HC	15	9.00	14.00	11.60	1.92	0.050
Object assembly	ED	15	3.00	16.00	9.53	3.54	
WISC-R	HC	15	7.00	17.00	12.00	2.88	0.070
Coding	ED	15	5.00	17.00	9.87	3.09	
PST	HC	15	0.00	9.00	3.13	2.77	0.007*
	ED	15	0.00	7.00	1.13	1.77	
PST strategy	HC	15	0.00	5.00	2.27	1.67	0.011*
	ED	15	0.00	6.00	1.00	1.51	
PST process	HC	15	0.00	21.00	7.13	6.14	0.006*
	ED	15	0.00	16.00	2.67	3.98	
CSEI	HC	15	24.00	42.00	35.87	5.78	0.517
	ED	15	28.00	46.00	35.07	5.39	
RCADS-CV	HC	15	27.00	85.00	44.93	16.05	0.236
	ED	15	35.00	74.00	48.87	12.15	
CBCL	HC	15	4.00	85.00	38.73	22.42	0.319
	ED	15	16.00	100.00	48.06	22.40	

HC, Healthy Control; ED, Endocrine Disorder. Test of Language Development-Primary: Fourth Edition Turkish Revision (TOLDP-4:T), Turkish Nonword Repetition Test (TNRT), The Turkish Multilingual Sentence Repetition Test (LITMUS-TR), Wechsler Intelligence Scale for Children (WISC-R), Problem-Solving Test (PST), The Revised Child Anxiety And Depression Scale – Child Version (RCADS-CV), Coopersmith Self-Esteem Inventory (CSEI), Child Behavior Checklist (CBCL), Wechsler Intelligence Scale for Children (WISC-R), Problem-Solving Test (PST); Mann–Whitney U Test, *p* < 0.05*.

The PST is highly positively correlated with verbal language score (*r* = 0.659, *p* = 0.001) and TOLDP-4:T sentence repetition (*r* = 0.688, *p* = 0.001). Using PST strategy is highly positively correlated with TOLDP-4:T verbal language score (*r* = 0.654, *p* = <0.001) and TOLDP-4:T sentence repetition (*r* = 0.686, *p* = <0.001). The PST process is highly correlated with TOLDP-4:T verbal language score (*r* = 0.647 *p* = <0.001) and TOLDP-4:T sentence repetition (*r* = 0.716, *p* = <0.001). Picture completion was moderately positively correlated with the PST strategy (*r* = 0.521, *p* = 0.003) and the PST process (*r* = 0.464, *p* = 0.010).

In Table 4, the results of the Mann–Whitney U Test for descriptive statistics and a comparison of the utilization of PST strategy and process used in the study of participants with endocrine disease and healthy development are presented. When groups are compared, draw a diagram (*p* = 0.0004), reason (*p* = 0.1327), find correlations (*p* = 0.0293), simplify the problem (*p* = 0.0141), guess and check (*p* = 0.5170), make a table (*p* = 0.3259), formulate (*p* = 0.0118), execute (*p* = 0.0441), and interprete and evaluate (*p* = 0.0458).

**Table 4:** Comparison of participants’ Problem-Solving Test subtests performances.

	Group	*N*	Min	Max	Mean	SD	*p*
Draw a diagram	ED	15	0	3	0.13	0.35	0.0004*
	HC	15	0	9	0.73	0.46	
Logical reasoning	ED	15	0	10	0.67	0.72	0.132
	HC	15	0	18	1.13	0.92	
Look for a pattern	ED	15	0	1	0.07	0.26	0.029*
	HC	15	0	8	0.53	0.74	
Simplify the problem	ED	15	0	1	0.07	0.53	0.014*
	HC	15	0	8	0.26	0.64	
Guess and check	ED	15	0	2	0.13	0.52	0.517
	HC	15	0	4	0.27	0.59	
Make a table	ED	15	0	1	0.07	0.26	0.325
	HC	15	0	0	0.00	0.00	
Make a systematic list	ED	15	0	0	0	0	0
	HC	15	0	0	0	0	
Use variable	ED	15	0	0	0	0	0
	HC	15	0	0	0	0	
Working backwards	ED	15	0	0	0	0	0
	HC	15	0	0	0	0	
Formulation	ED	15	0	8	0.53	0.83	0.011*
	HC	15	0	26	1.87	1.73	
Execution	ED	15	0	19	1.27	2.02	0.044*
	HC	15	0	47	3.13	2.77	
Interpretation and evaluation	ED	15	0	13	0.87	1.30	0.045*
	HC	15	0	30	2.00	1.65	

HC, Healthy Control; ED, Endocrine Disorder. Mann–Whitney U Test, p < 0.05*..

### Problem-Solving Test: qualitative data analysis

In this section, the participants’ responses to the questions of the Problem-Solving Test are elaborated through strategies. Participants ED-4 and ED-13 could not answer any of the questions correctly. Therefore, the relevant participants were not included in the strategy explanations.

#### Strategy 1: “Make a Systematic List”

*Item 1: A hacker who wishes to decrypt a computer program discovers that the ciphers are groups of numbers that allow the number 20 to be obtained using 8 odd numbers. What are the different ciphers that use 8 odd numbers to get 20?* (Temel and Altun, 2020).

HC-1, HC-2, and HC-6 reasoned according to the odd number rule, but they failed to use the Make a Systematic List strategy. HC-4 and HC-7 attempted to perform arithmetic operations with numbers that were not by the rules but were specified in the problem statement, but failed to use the strategy and could not reach the correct result. HC-5 reasoned in line with the odd number rule, added and subtracted two numbers with their odd sum being 20, but did not realize that he had to use 8 odd numbers and could not reach the correct result. HC-10, HC-12, and HC-15 drew eight lines and left them. They did not do anything per the requirements of the problem and did not reach the correct result.

ED-3 drew eight lines, wrote a password on them, and followed the rule of the problem that the sum of the numbers in the password should be 20, but he did not apply the odd number rule and could not reach the correct result. ED-5 wrote 3 different ciphers, followed the odd number rule while writing these ciphers, but did not follow the rule that the sum of 8 numbers should be 20, found one of the ciphers correct, but could not find all of the ciphers and could not reach the correct result. ED-6 wrote 8 odd numbers separated by commas, followed the odd number rule, and was aware that there should be 8 numbers but did not pay attention to the fact that the sum of the numbers should be 20 and could not reach the correct result. ED-8 performed arithmetic operations that did not comply with the rules of the question, did not specify any result, or could not reach the correct result. He performed arithmetic operations with the numbers in the question that were not under the rules. He subtracted 8 from 20, found 12, and expressed it as 1–2, but could not reach the correct result. ED-10 did not list any possible situations in the question, did not make mathematical assumptions in line with the odd number rule, wrote the numbers from 1 to 6 and left them, and could not reach the correct result. ED-12 listed two situations in the question but did not perform a systematic ordering, made reasoning with mathematical assumptions in line with the odd number rule, performed arithmetic operations, and could not reach the correct answer because he did not write all the situations.

*Item 10: How many different total points does a person who shoots three times at the shooting plate in the figure get if he hits the targets each time?* (Temel and Altun, 2020).

HC-2 and HC-7 attempted to perform arithmetic operations with the numbers in the question, which were not in accordance with the rules and could not reach the correct answer. HC-3 and HC-5 wrote numbers directly without performing any arithmetic operations or using a list and could not achieve the correct result. HC-6 placed points on the targets, but he did not provide a result about how many different points could be obtained.

ED-1 gave different scores to three target parts of the plate and could not reach the correct conclusion by inferring that a total of three different scores could be obtained. ED-3 wrote a direct answer without any processing and could not reach the correct conclusion. ED-9 multiplied 12 by 3 to obtain 36 and was unable to reach the correct conclusion. ED-12 did not make a systematic listing, did not make assumptions about which points could be scored, did 5×3 = 15, deleted the 5×3 part and left it as 15, and could not reach the correct answer.

#### Strategy 2: “Guess and Check”

*Item 2: Place the numbers 1, 2, 3, 4, 5, and 6 in the circles shown in the figure so that the sum of the numbers on the sides of the triangle formed by the circles is 9* (Temel and Altun, 2020).

HC-1, HC-2, HC-3, and HC-4 guessed and filled in the circles but could not make arithmetic calculations or use the estimation and control strategy. HC-5 guessed and filled in the circles, checked, erased, and rewrote, but arranged the circles so that each side was 12 instead of 9 and could not reach the correct result. HC-6 guessed, filled in the circles, checked, erased, and rewrote, but arranged only 2 sides as 9 and was unable to reach the correct result. HC-7 and HC-10 tried to place the given numbers inside the circles but could not arrange them in such a way that the sum of all sides was 9 and could not reach the correct result. HC-8 wrote 9 in the round in each corner, did not follow the given rules, and could not reach the correct result. HC-11 and HC-14 placed the numbers inside the circles but did not follow the rule of 9 on only two sides and could not reach the correct result. HC-12 and HC-15 placed the numbers inside the circles, made arithmetic calculations, and reached the correct result.

ED-1 attempted to place the numbers inside the circles but failed to fulfill the rule that the sum of the sides should be 9 and could not reach the correct result. ED-5 and ED-7 attempted to place numbers in the circles but did not follow the rule that one side should be 9, and could not reach the correct result. ED-8 attempted to place the numbers in the circles, could only follow the rule that one side should be 9 on 2 sides, and could not reach the correct result. ED-9 placed the given numbers except four in the circles and made arithmetic calculations in such a way that the sum of two sides would be nine, placed the number nine in the empty round on one side, and could not reach the correct result. ED-10 placed the given numbers in the circles, did not pay attention to what was asked in the question, did not perform arithmetic calculations for any of the sides that did not equalize to nine, and could not reach the correct result. ED-11 placed the given numbers in circles, did not pay attention to what was asked in the question, did not perform any arithmetic operations for any side that did not equalize to nine, and could not reach the correct result. ED-12 made assumptions about the solution to the problem, filled in the circles, performed arithmetic calculations, checked that each side was 9, and reached the correct answer. ED-14 made assumptions regarding the solution of the problem, filled the circles only with the numbers one and six, verified that the two sides were nine by doing arithmetic calculations, and could not reach the correct answer.

*Item 11: The quiz team from class 8-A scored 44 points by correctly answering 12 questions of 3 or 5 points. How many 5-point questions did the quiz team answer correctly?* (Temel and Altun, 2020).

HC-2 and HC-7 tried to do arithmetic operations with the numbers in the question, which were not in accordance with the rules and could not reach the correct result. HC-3 used the strategy by making logical inferences by making assumptions about the solution to the problem mentally and reaching the correct result. HC-5 and HC-6 wrote numbers directly without any arithmetic operations, guessing, or checking and could not reach the correct result. HC-15 estimated how many points could be obtained from 12 questions, made arithmetic calculations, made logical inferences, and reached the correct answer.

ED-1, ED-3, and ED-6 wrote an incorrect answer without doing any operations or drawing a diagram and could not reach the correct answer. ED-12 made several different assumptions, tested these assumptions, and inferred that he did not make the correct assumption. He finally reached the correct result by writing the answer 8*a = 24 and a = 3 and giving the correct answer to four questions.

#### Strategy 3: “Draw a Diagram”

*Item 3: A frog located at the bottom of a 9-meter-deep well is struggling to get out of the well. With each jump, it rises 4 meters and then slides back 1 meter because the wall is slippery. At what jump does the frog get out of the well?* (Temel and Altun, 2020).

HC-1 and HC-3 conducted and erased operations and reached the correct conclusion without drawing a diagram. HC-2 performed arithmetic operations not in accordance with the rules, could not draw a diagram, and could not reach the correct result. HC-4 performed the arithmetic operations in line with the instructions related to the problem and reached the correct result. HC-5 concluded that he would rise 9 meters in the 3rd jump, but he could not reach the correct result because he believed that he would come out of the well completely in the 4th jump. HC-6 provided a direct response by conducting mental calculations and reached the correct result. HC-7 attempted to perform arithmetic operations with the numbers in the question, which were not in accordance with the rules, multiplied 9 by 4, and could not reach the correct result. HC-12, HC-13, and HC-15 recognized the 4–1 pattern based on the figure they had drawn and reached the correct result accordingly.

ED-1 did not draw a diagram, recognized the 4–1 pattern, inferred that she could exit on the 3rd jump, and reached the correct conclusion. ED-3 tried to draw a diagram, noticed the 4–1 pattern, first wrote 4 and then shifted it to 3, wrote 7 and shifted it to 6, but made one more step after 9, wrote that it was the 5th jump, not the 4th jump, and could not reach the correct answer. ED-5 wrote 9,4,1 in a row, made a logical inference from the mind, and wrote 3 times 3 meters of jumping and reached the correct answer. ED-7 did not draw any shapes, did not perform any operations, wrote 4 directly on it, and could not reach the correct result. ED-8 and ED-9 noticed the 4–1 pattern but did not continue it. After finding 4–1 = 3, they subtracted 3 from 9 and inferred that they could get out of the well on the 6th jump, but could not reach the correct conclusion. ED-10 did not perform any operation in line with any drawing and the instructions in the question, did not notice the mathematical pattern and structure, wrote the same answer he wrote in the 1st question, and could not reach the correct result. ED-11 did not make any drawings and did not take any action in line with the instructions in the question, did not notice the mathematical pattern and structure, wrote 2 in the answer, and could not reach the correct result. ED-12 made a drawing, noticed the mathematical pattern of 4–1 in line with the instructions, and wrote that he/she could exit on the 3rd jump and reach the correct result.

*Item 12: Erkin has a plastic toy train with a circular track. There are 6 stations around this track at equal distances from each other. The train travels from station 1 (the starting station) to station 3 in 12 s. At the same speed, how long does it take the train to complete one lap?* (Temel and Altun, 2020).

HC-2, HC-4, and HC-7 tried to do arithmetic operations with the numbers in the question, which were not in accordance with the rules, but could not reach the correct result. HC-3 wrote a number directly without any arithmetic operation and without using a list and could not reach the correct result. HC-5 wrote a direct number without doing any arithmetic operation or using a list and could not reach the correct result. It is thought that he tried to reach the result by calculating five intervals. HC-6, without drawing a diagram, thought that if it took 12 s until the 3rd station, it took 24 s until the 6th station and could not reach the correct result. HC-12 and HC-15 reached the correct result by drawing a diagram and determining the distance between the stations.

ED-1 and ED-3, without drawing a diagram, thought that if it took 12 s until the 3rd station, it would take 24 s until the 6th station and wrote a direct answer without processing and could not reach the correct result. Without drawing a diagram, ED-5 thought that if it took 12 s until the 3rd station, it would take 12 s for 3 more stations. It made 12 + 12 = 24 and could not reach the correct result. He drew 6 linear stations and wrote 12 under the 2nd station, 3–5. ED-12 wrote 12 between stations 3–5 and 5–6. He wrote 6 between the stations, added the 3 numbers, and found the answer 30, but since he did not draw a circular diagram, he missed between the stations and could not reach the correct result.

#### Strategy 4: “Look for a Pattern”

*Item 4: 10 bricks were used for the construction of the 4-step staircase given in the figure below. It is desired to build a staircase with 15 steps as shown in the figure. How many bricks are necessary for the new staircase?* (Temel and Altun, 2020).

HC-1 tried to draw a diagram, tried to complete the stairs, and failed to reach the correct result by counting the bricks incorrectly. HC-2 attempted to do arithmetic operations with the numbers in the question, which were not in accordance with the rules, could not establish a relationship, and could not reach the correct result. HC-3 and HC-12 inferred the number of bricks on which digit, performed arithmetic operations, erased, and arrived at the correct result. HC-4 performed a drawing to solve the problem and realized how many bricks were on which step, but performed incorrect operations and could not reach the correct result. HC-5 could not reach the correct result by writing a number directly without performing any operation or finding a pattern. HC-6 drew a picture with 5 bricks on the 5th step, concluded that 15 bricks would form 5 steps, failed to realize what was asked in the question, and could not reach the correct result. HC-7 added 10 and 5, could not reach the correct result, thought that there were 5 bricks in the 5th step, could not understand the difference between 15 bricks and 15 steps, and could not realize what was asked in the question. HC-9 completed the picture with his drawing and realized that there were 1…0.1…0.7…0.7 bricks on the 1st step, and then counted the bricks he drew and reached the correct result.

ED-1 drew the stairs from the beginning, drew the correct number of bricks in small numbers of steps, confused the number of bricks as the number of steps increased, then stopped drawing, wrote two numbers as 30 and 34 without any operation, and was unable to reach the correct result. ED-2 drew to increase the number of steps by one but did not draw the rest, did not write any numbers, and could not reach the correct result. ED-3 drew another step in the continuation of the stairs, did not separate the bricks, wrote 5 bricks as an answer, and realized that there were 15 bricks in total, but did not realize that the question asked how many bricks were needed for 15 steps and could not reach the correct result. ED-6 wrote 5 bricks without doing any operation or drawing a picture and could not reach the correct answer. ED-8 and ED-9 multiplied 15 because 15 digits were asked and 10 because they saw 10 bricks, and they did the arithmetic operation incorrectly and could not reach the correct result. ED-11 did not make any inferences about the problem, did not notice the mathematical pattern, did an arithmetic calculation multiplied 15 by 4 to get 60, and could not reach the correct result. ED-12 drew a diagram of a staircase with 6 steps and left it, realizing that there should be 1 brick for the 1st step and 15 bricks for the next 15 steps. ED-12 realized the mathematical pattern, made arithmetic calculations, and reached the correct result.

*Item 13: The length of one side of the regular hexagon shown in Figure 1 is 1 cm. The length of the perimeter of two hexagons joined side by side in Figure 2 is 10 cm, and the length of the perimeter of 3 hexagons joined side by side in Figure 3 is 14 cm. If we were to connect 7 hexagons as shown in the figures, what would be the perimeter length of the resulting shape?* (Temel and Altun, 2020).

HC-1 wrote numbers without any operation and could not find a relation. HC-3 mentally noticed the order and relationship of the problem, wrote the result as a number, and reached the correct answer. HC-5 believed that since the perimeter of the union of 3 hexagons was 14 cm, the perimeter of the union of 6 hexagons would be 28, and when 1 more hexagon was added, one side would not be counted and inferred that the perimeter of the union of 7 hexagons would be 33 cm. HC-6 drew 4 more hexagons next to 3 hexagons, counted their sides, and reached the correct answer. HC-7 attempted to perform arithmetic operations with the numbers in the question, which were not in accordance with the rules, did not understand what was asked in the problem, found the number of hexagons, could not use the strategy, and did not reach the correct result. HC-10 drew a picture, counted the total number of sides, and reached the correct result. HC-12 drew a figure, performed arithmetic operations on the figure, and reached the correct result. HC-15 calculated the perimeter of 7 hexagons, performing arithmetic operations by subtracting their intersections, and achieving the correct result.

ED-1 drew 4 more hexagons, counted their sides incorrectly, reached the result 29, and could not answer correctly. ED-3 stated that it was 14 cm under 3 hexagons, drew 4 additional hexagons next to it, counted the perimeter incorrectly, and could not reach the correct result. ED-5 wrote a direct answer without drawing any shape or doing any operations and could not reach the correct answer. ED-10, as he did in Questions 1 and 3, wrote from 1 to 6 and left it, did not notice any mathematical structure or correlation with the problem, and could not reach the correct result.

#### Strategy 5: “Use a Variable”

*Item 5: Kübra’s new bicycle has a device on the steering wheel that measures average speed. Kübra decides to ride her bicycle to her aunt in another neighborhood. When Kübra arrives at her aunt’s house, the device shows an average speed of 8 km/h, and on the way back, it shows an average speed of 10 km/h. Since the return trip takes 4 h, how long is Kübra’s travel time?* (Temel and Altun, 2020).

HC-1 tried to make arithmetic calculations without equations and failed to use the strategy of using variables. HC-2 and HC-12 tried to perform arithmetic operations with the numbers in the question, which were not in accordance with the rules, could not use variables, and could not reach the correct result. HC-3, HC-4, HC-5, and HC-6 wrote an answer without doing any arithmetic operations or equations, did not use variables, and could not reach the correct result. HC-7 attempted to perform arithmetic operations with the numbers in the question, which were not in accordance with the rules, could not use the strategy of multiplying two rates by each other, and could not reach the correct result.

ED-1 wrote a direct answer without considering the instructions and could not reach the correct result. ED-3 wrote a direct number without doing any operation and could not reach the correct result. ED-5 attempted to do arithmetic operations that were not in accordance with the rules, could not use variables, and could not reach the correct result. ED-8 did arithmetic operations with the numbers in the question that were not in accordance with the rules. They added 8 and 10 and subtracted 4 from the result to get 14, and could not reach the correct result. ED-9 did arithmetic operations with the numbers in the question that were not appropriate for the problem, divided 4 by 2, and found 2 and could not reach the correct result. ED-12 did not use any equation, wrote that 1 h is 60 min and 4 h is 240 min, multiplied 24 by 8 and divided the result by 60, and wrote 3 h and 12 min on another side and could not reach the correct result.

*Item 14: The sum of three numbers proportional to the numbers 5, 7, and 11 is 207. Find each number* (Temel and Altun, 2020).

HC-2 tried to do arithmetic operations with the numbers in the question, which was not in accordance with the rules and could not reach the correct result. HC-7 tried to do arithmetic operations with the numbers in the question, but they were not in accordance with the rules, could not use the strategy, and could not reach the correct result. HC-11 wrote the questions to be proportional, but he did not perform any further operations and could not reach the correct result. HC-13 tried to build a mathematical structure by adding three proportions but stopped halfway and could not reach the correct result.

ED-3 drew three boxes, one under the other, added them, and wrote 207. He gave values to the boxes as 100,100,7, and left them, but could not reach the correct result. ED-10 wrote the numbers side by side, failed to formulate any mathematical structure related to the problem, and could not reach the correct conclusion.

#### Strategy 6: “Simplify the Problem”

*Item 6: Ayşe bought boxes from a toy shop to put her toys in boxes. There are two medium-sized boxes in each of the large boxes and two small-sized boxes in each of the medium-sized boxes. Since Ayşe bought 5 large boxes from this shop, how many boxes did Ayşe have in total?* (Temel and Altun, 2020).

HC-1 and HC-4 did not make any simplifications or generalizations and could not use the strategy. HC-2 tried to do arithmetic operations with the numbers in the question, which were not in accordance with the rules, did not simplify, and could not reach the correct result. HC-3 did the simplification mentally and wrote the correct result directly. HC-5 wrote the answer without doing any operation or drawing a diagram and could not reach the correct result. HC-6 drew a diagram, erased it, wrote the number directly, and could not reach the correct result. HC-7 tried to do arithmetic operations with the numbers in the question, multiplied 5 and 2, but failed to use the strategy and could not reach the correct result. The HC-9, HC-10, HC-12, and HC-15 calculated the total number of boxes by drawing, generalized it, and multiplied it by 5. They then used the simplification and strategy to reach the correct result.

ED-1 drew a correct diagram by paying attention to the instructions of the question, failed to simplify and generalize it, counted the boxes incorrectly, and did not reach the correct result. ED-3 drew 8 nested boxes and wrote 9 next to them, did not draw a diagram following the rules in the question, did not operate, and could not reach the correct result. ED-5 did not draw any diagram, multiplied 5 by 3, and found 15 and could not reach the correct result. ED-6 did not execute any operations, did not draw a diagram, directly wrote the answer as 9 boxes, and could not reach the correct result. ED-8 did not draw any diagram, did the operation 2 + 2 + 5 = 8, and did not reach the correct result. ED-9 wrote 4 twos one after the other and left the sum as 8 and could not reach the correct result. ED-11 drew any shape, did not simplify, did not generalize, wrote the answer 9 without any operation, and could not reach the correct result. ED-12 drew and counted 5 large boxes, 2 medium-sized boxes under each of them, and 2 small-sized boxes under each of them, 35 boxes in total, and reached the correct result. Instead of drawing 35 boxes and then simplifying, generalizing, and processing them, he drew each one individually.

*Item 15: Place the numbers from 1 to 19 inside the 19 circles given below in such a way that the sum of every 3 numbers in a direction gives the same result (The numbers marked in bold in the figure represent the direction)* (Temel and Altun, 2020).

HC-1 tried to place the numbers in one direction but left the other directions empty. He could not continue the execution process successfully. HC-4 tried to do arithmetic operations with the numbers in the question, which were not in accordance with the rules but could not reach the correct result. HC-5 tried to match the numbers in the desired direction and simplified the problem by dividing the numbers by 2 and reaching the correct result. HC-6 tried to write the numbers in circles, could not match the numbers in the direction, skipped the number 3, and wrote the number 17 in 2 circles, and could not reach the correct result. HC-7 tried to do arithmetic operations with the numbers in the question, which were not in accordance with the rules, could not use the strategy, and could not reach the correct result. HC-11 tried to match the numbers in the direction, simplified the problem by dividing the numbers by 2, and reached the correct result. HC-12 simplified the problem by trying to match the numbers in the direction of dividing the numbers by 2 and reaching the correct result.

ED-3 wrote numbers in three circles in only 1 direction, omitted them, and did not reach the correct result. ED-6 wrote numbers from 1 to 19a inside the circles in order but did not follow the rule that the sums of the numbers in all directions should be equal and did not reach the correct result. ED-7 wrote the numbers 1 to 19a inside the circles in order, did not follow the rule that the sums of the numbers in all directions should be equal, and could not reach the correct result. ED-10 scribbled inside the circles, could not interpret the instructions related to the problem correctly, did not make any simplification or arithmetic operation, and could not reach the correct result.

#### Strategy 7: “Working Backwards”

*Item 7: When paying the bill, a restaurant owner tells his customers, “Look inside the cash register, put as much money as you have, take 2 liras and leave.” The fourth customer looks in the cash register and sees that there is no money. How many liras were in the cash register before the customers?* (Temel and Altun, 2020).

HC-1 and HC-6 wrote numbers without any operation and did not reach the correct result. HC-4 tried to do arithmetic operations with the numbers in the question, which is not in accordance with the rules and could not reach the correct result. HC-7 tried to do arithmetic operations with the numbers in the question without following the rules, multiplied 2 by 8, failed to use the strategy, and could not reach the correct result.

ED-3, ED-9, and ED-10 wrote a direct answer without any operation and could not reach the correct result.

*Item 16: A plastic ball dropped from a height rises 3/5 of the height from which it was dropped each time. Since it rises 81 cm on the fourth bounce, from how many meters high was the ball dropped?* (Temel and Altun, 2020).

HC-1, HC-2 HC-7, and HC-7 tried to do arithmetic operations without following the rules and algorithm and could not use the backward working strategy. HC-6 did not use any operation, wrote a direct answer, and could not reach the correct result. HC-12 tried to apply the given algorithm from the 5th jump to the beginning but did not solve the problem until the 2nd jump did not continue the problem and could not reach the correct result.

ED-3 tried to apply the algorithm backward from the 5th jump, divided 81 into 3, and left the process there, did not continue, and could not reach the correct result. ED-5 did not perform the operation by the rules, did not follow a logical path, performed an arithmetic operation, found 81×5 = 405 cm, converted it to meters since the question asked from how many meters high, wrote it as 4 m 5 cm, and could not reach the correct result. ED-9 did arithmetic operations with numbers that were not in accordance with the rules and could not reach the correct result. ED-10 did not realize what the problem asked, did not apply the algorithm for the problem, wrote “20 balls were dropped,” and could not reach the correct result. With the answer he gave, it is assumed that he counted the points on the lines in the visual of the problem. ED-12 could not apply the given algorithm, concluded 16 + 16 = 32 81 + 32 = 113, and could not reach the correct result.

#### Strategy 8: “Make a Table”

*Item 8: A carpenter makes stools with 3 legs and tables with 4 legs. If 31 legs are used at the end of a day, how many tables and how many stools can he have made that day?* (Temel and Altun, 2020).

HC-1 found a situation correct by guessing and checking but was incomplete because he did not make a table and could not execute. HC-2 tried to do arithmetic operations with the numbers in the question, which was not in accordance with the rules, could not use the strategy of making a table, and could not reach the correct result. HC-4 tried to do arithmetic operations with the numbers in the question, which were not in accordance with the rules and could not reach the correct result. HC-5 did not use the “Make a Table” strategy, wrote only one of the possibilities correctly without using any operation, and could not continue. HC-7 tried to do arithmetic operations with the numbers in the question, which were not in accordance with the rules, added and multiplied the numbers with each other, failed to use the strategy, wrote what he did wrong, and could not reach the result. HC-10 wrote the numbers in the problem one after the other and could not reach any result. HC-12 found 1 possibility correct in line with the given data, added the number of tables and stools, and could not reach the correct answer.

ED-1 guessed 3 tables and 5 stools, then guessed 4 tables and 4 stools, reached a conclusion without paying attention to the rules, and could not get it right. ED-3 assigned titles of table and stool, found the possibility of 7 tables and 1 stool correct, could not guess the other possibilities, could not complete the question, and could not reach the correct result. ED-5 made an assumption and subtracted 16 from 31 (it is thought that 16 is calculated as 4 table legs), divided the remaining number by 3, and reached the result of 5 stools, but found only 1 case and could not continue the rest because he did not draw a table and could not reach the correct result. ED-6 multiplied 31 by 4 and 3 separately, without following the rules, and summed the results and found 217, but could not reach the correct result. ED-9 divided the number 31 feet by 2 and wrote chair next to 16 and stool next to the remaining 1 and could not reach the correct result. ED-10 wrote 1,2,3,4 in the order of 1,2,3,4 as he wrote in the previous questions without making any inferences or creating a table in line with the given information and could not reach the correct result. ED-12 did not make a mathematical table, drew 31 lines and divided them into groups of 3 and 4, inferred 5 stools and 4 tables, could not calculate other possibilities, and could not reach the correct result.

*Item 17: A company gives a bonus of 5 liras to its salesmen who sell between 5 and 10 products, 2 liras for each product they sell more than 10, and no bonus to those who sell less than 5. At the end of the day, how many products did a seller who received a bonus of 11 liras sell that day?* (Temel and Altun, 2020).

HC-1 tried to do arithmetic operations without following the rules and without making a table and could not reach the correct result. HC-5 and HC-6 wrote a direct answer without doing any operations and did not reach the correct result. HC-12 wrote what was given but did not perform any operations on them and could not reach the correct result.

ED-3 did not perform any operation, wrote the answer directly, and could not reach the correct result. ED-10 did not make any table, did not make any inference from the given information about the problem, and when the question asked for the number of products, E-10 wrote “sold 50 liras” and could not reach the correct result. ED-11 did not make any table, did not make any inferences from the given information about the problem, wrote 5,6, and could not reach the correct result. ED-12 did not make a table in line with the given data, but did the operation 5 + 2 + 2 + 2 + 2 = 11, wrote 10 over 5, 11, 12, 13 over 2 s, respectively, and reached the correct result by adding the result “sold 13 products.”

#### Strategy 9: “Logical Reasoning”

*Item 9: Five cars have participated in a race. The numbers on the cars participating in the race are as follows:*

*1733 5824 9762 6465 7525*

- *The car with the biggest number finished the race last.*
- *The 1st car and the 2nd car have the same sum of digits in their digits.*
- *The number in the ones digit of the 3rd and 4th cars is odd.*
- *The numbers of the 2nd and 3rd cars are divisible by 5.*

*According to the given information, in which order did the cars finish the race?* (Temel and Altun, 2020).

HC-1 could not evaluate the outputs related to the problem correctly and could not conclude. HC-2, HC-3, HC-4, HC-5, HC-10, HC-12, HC-13, HC-14, and HC-15 used the reasoning strategy by evaluating the mathematical outcomes of the problem and reached the correct conclusion. HC-6 and HC-7 correctly evaluated the mathematical outputs related to the problem, such as finding the largest number, incorrectly evaluated the sum of digits, incorrectly evaluated the rule of division by 5, and could not reach the correct conclusion.

ED-1 correctly interpreted the largest number, the number in the one’s digit being odd, incorrectly interpreted the addition of digits and the rule of divisibility by 5, and could not reach the correct conclusion. ED-3 correctly interpreted the instruction with the largest number, correctly calculated the sums of digits in the digits but could not put them in the correct order, correctly interpreted the odd number rule but could not put them in the correct order, incorrectly interpreted the rule of divisibility by 5 and could not reach the correct conclusion. ED-5 correctly interpreted the instruction with the largest number and put it in the 5th place. It correctly interpreted the instruction that the sum of the numbers in the digits is equal but did not specify the order of the cars according to the remaining instructions and could not reach the correct conclusion. ED-6, ED-7, and ED-12 used the reasoning strategy by evaluating the mathematical outputs related to the problem and reached the correct conclusion. ED-9 correctly interpreted and found the greatest number rule, but did not make any evaluation of the instructions at the end, left the problem unfinished, and did not reach the correct conclusion. ED-10 could not evaluate the outputs related to the problem correctly, did not make any determination, reasoning, or inferences, only wrote 9,762 and left it, and could not reach the correct conclusion.

*Item 18: Three runners named İlker, Naci, and Alper are running toward the stadium. Ilker is always correct. Naci is sometimes correct. Alper is never correct. Identify the names of the runners* (Temel and Altun, 2020).

HC-1, HC-2, HC-3, HC-4, HC-5, HC-6, and HC-7 went through an evaluation process for the outputs related to the problem but did not reach the correct conclusion. HC-10, HC-11, HC-12, HC-13, HC-14, and HC-15 went through an evaluation process in line with the outputs given in the problem and reached the correct conclusion.

ED-1, ED-6, and ED-14 could not interpret the outcomes of the problem correctly and could not reach the correct conclusion. ED-3, ED-5, ED-7, ED-8, ED-11, ED-12, and ED-15 had an evaluation process in line with the outcomes of the problem and reached the correct conclusion. ED-10 could not interpret the outcomes of the problem correctly, wrote 2,1,3 under the runners in order while asking question names, and could not reach the correct conclusion.

#### Total strategy

During problem solving, “Draw a Diagram,” “Look for a Pattern,” “Simplify the Problem,” “Guess and Check,” “Use a Variable,” and “Logical Reasoning” are the strategies seen in the healthy control group. During problem solving, “Draw a Diagram,” “Look for a Pattern,” “Simplify the Problem,” “Make a Table,” “Make a Systematic List,” “Use a Variable,” “Logical Reasoning,” and “Working Backwards” patterns were the strategies seen in the group of individuals with endocrine disorders.

#### Processes

During problem solving, formulating, interpreting, and executing are the processes seen in the group of healthy controls and individuals with endocrine disorders.

All results were examined by two independent speech-language pathologists at different times; children with healthy development were able to “Guess and Check,” “Draw a Diagram,” “Use a Variable,” “Simplify the Problem” and “Logical Reasoning” strategies but were not able to “Make a Systematic List,” “Use a Variable,” “Working Backwards,” and “Make a Table” strategies at all; children with endocrine disease were able to “Guess and Check,” “Draw a Diagram,” “Look for a Pattern,” “Simplify the Problem,” “Make a Table,” and “Logical Reasoning,” strategies, but could not “Make a Systematic List, “Use a Variable,” and “Working Backwards” strategies at all; except for E12, children with endocrine disease could not use any strategy except “Draw a Diagram” “Logical Reasoning” strategies; 10 (66.6%) of the children with healthy development were able to use all formulation, execution, interpretation and evaluation processes while using strategies; 2 children with endocrine disease (13%) were able to use all of the formulation, execution, interpretation and evaluation processes while using the strategies; children with healthy development answered a total of 47 questions (17%), while children with endocrine disease answered a total of 18 questions (6%); 13. Hexagon joining; it was observed that only children with healthy development (33.3%) answered correctly the question that could be answered using the strategy of “Look for a Pattern”; 12. Toy Train; it was observed that only children with healthy development (13.3%) answered correctly the question that could be answered using the strategy of “Draw a diagram”; 15. Circle Filling; it was observed that only children with healthy development (20%) answered correctly the question that could be answered using the strategy of “Simplify the Problem”; and 17. Premium; it was observed that only children with endocrine disease (6.6%) answered correctly the question that could be answered using the strategy of “Make a Table.”

## Discussion

In this study, children with endocrinologic diseases were compared with their peers with normal development in terms of language, cognition, mathematical skills, and psychosocial characteristics. The data obtained from the study showed that children with endocrine diseases showed lower performance in language, cognition, and mathematical skills compared to their healthy peers, while they did not differ in terms of social–emotional status assessed by psychological scales. In the literature, it has been reported that childhood chronic diseases negatively affect language and cognitive functions, and difficulties may arise in the areas of academic skills and social–emotional development due to the direct and indirect effects of medical factors associated with the disease (Jones et al., 2017; Forrest et al., 2011). Consistent with the literature, children with endocrine diseases in the current study showed lower performance in language, cognition, and mathematical skills compared to their healthy peers.

The quality of life of the person living with chronic disease is affected in terms of physical, mental, and social functioning (Verbrugge et al., 1989). Therefore, having a chronic disease increases the risks of social isolation, loneliness, inadequacy, fatigue, anger, hopelessness, frustration, anxiety, and depression (Gerontoukou et al., 2015). Similarly, mood disorders such as anxiety, depression, and mania have been reported in various childhood endocrine diseases and it has been suggested that quality of life may decrease (Sonino et al., 2015). Self-concept is a psychological construct formed through interpersonal experiences in social contexts and influenced by genetic endowment and the expectations and judgments of significant others (e.g., parents, siblings, peers) (Bong and Skaalvik, 2003). Despite extensive meta-analysis data showing that self-confidence (Pinquart, 2013) and self-perception are negatively affected in children with chronic diseases compared to their healthy developing peers, it remains a challenge to reach clear conclusions due to sample size and methodological inadequacies (Ferro and Boyle, 2013). In this study, no difference was found between children with endocrine diseases and their healthy peers in terms of depression, self-esteem, and the presence of behavioral problems.

The lower TOLDP-4:T scores of children with endocrinological disorders compared to their healthy peers indicate that hormonal disorders negatively affect listening, organizing, speaking, grammar, and semantic components of language; poor TNRT scores indicate limited phonological working memory capacity; WISC-R scores indicate lower cognitive functions; and PST scores indicate inadequate mathematical performance. When WISC-R subtests were compared, it was found that children with the endocrine disease showed lower performance in total verbal performance, information, comprehension, arithmetic, similarities, vocabulary, total performance, picture completion, and picture arrangement scores compared to the control group.

Verbal mathematical problems, which are considered routine and non-routine problems, are mathematical tasks in which the information required for the solution is presented as text instead of mathematical notation and can range from verbal descriptions of basic arithmetic operations to advanced modeling problems. In routine mathematical problems, there is a linear syntax, and the solution is reached by deducing the necessary steps and performing arithmetic operations. In non-routine problems, on the other hand, determining the steps required for the solution requires the processing of complex syntactic structures and contextual elements, and abstract representations need to be produced to reach the solution (Strohmaier et al., 2022). The most commonly reported strategies in the literature for solving non-routine problems are “Draw a Diagram,” “Look for a Pattern,” “Simplify the Problem,” “Make a Systematic List, “Use a Variable,” “Logical Reasoning,” and “Working Backwards.” These strategies should be used in non-routine problem solving and formulation, execution, and interpretation processes should be employed (Temel and Altun, 2020).

The “Draw a Diagram” strategy facilitates the organization of the complex data in the problem statement by placing them in drawings such as figures and diagrams, thus providing a clearer understanding of the unknowns. The strategy of “Logical Reasoning” requires recognizing regular and repetitive patterns in mathematical expressions and determining the rule of this pattern. The “Simplify the Problem” strategy makes it possible to see the solution more clearly by reducing the numerical values of the data or categorizing them with various labels (Temel and Altun, 2020). In problem solving, expressing contextual elements related to real life in the mathematical language (formulation), processing the data given in the problem to solve by using reasoning steps and performing appropriate arithmetic operations (execution), evaluating the accuracy of the result, and adapting it to daily life (interpretation and evaluation) are considered as mathematical processes (Tout and Gal, 2015).

Children with endocrine disorders used “Draw a Diagram,” “Look for a Pattern,” and “Simplify the Problem” strategies and formulation, execution, and interpretation processes less frequently than their healthy peers. However, “Make a Systematic List, “Use a Variable,” and “Working Backwards” strategies were not used in children with endocrinologic disease and healthy control groups. The data obtained in this study reveal that the development of various strategies necessary for non-routine problem solving and the ability to carry out the necessary procedures for problem solving may be impaired in childhood endocrinologic diseases.

With the use of representations such as symbols, drawings, models, graphics, and physical objects, it becomes possible to concretize what is given and desired in the problem, present the problem situation, and determine the necessary steps for the solution. Another type of representation created to overcome the problem situation is linguistic representation. Linguistic representations are expressed in writing or speech (Goldin and Shteingold, 2001). In the construction process of the linguistic representation, a series of calculations can be planned, and a solution can be realized by writing down all the known information about the problem (Anwar and Rahmawati, 2017). Similarly, in studies investigating the effect of writing during mathematical problem solving, written and verbal descriptions were analyzed, and it was seen that written and verbal representations were correlated with the number of problem-solving strategies students tried (Pugalee, 2004). In this study, in line with the literature, the overall score of the Problem-Solving Test, the problem-solving strategies, and the process skills used were highly correlated with the TOLDP-4:T verbal language score and the TOLDP-4:T sentence repetition score. This finding emphasizes the decisive role of language skills in solving non-routine problems. Problem-Solving Test overall score, problem-solving strategies used, and process skills were moderately correlated with the WISC-R performance section and picture completion. The WISC-R performance section consists of picture completion, picture arrangement, block design, object assembly, and coding subtests and measures cognitive skills such as attention, memory, processing speed, hand-eye coordination, as well as visual–spatial skills (D'Angiulli and Siegel, 2003). Picture completion performance, another of the WISC-R performance subtests, is a test that measures visual alertness (vigilance) and the ability to distinguish important details from unimportant details, requiring the child to determine what is missing in pictures of objects or events frequently encountered in daily life. In this way, it measures the ability to distinguish between necessary and non-essential details and requires concentration, visual organization, and visual memory functions (Frankel et al., 2000). Visual perceptual skills are critical in solving non-routine problems, but linguistic skills have a greater weight as the main determinant (Strohmaier et al., 2022). Therefore, the presence of language impairment in childhood endocrinological diseases necessitates a more detailed evaluation of cognition and mathematical skills.

Childhood endocrinological diseases negatively affect language, cognition, and mathematical skills. In addition, sub-skill types such as problem-solving skills, which constitute the basic building block of mathematical performance, developing solution strategies, and executing the necessary steps to reach a solution are negatively affected by childhood endocrinological diseases. The fact that childhood endocrinological disorders are accompanied by developmental language and cognitive impairment may indicate poor mathematical performance. In addition, there are problem-solving strategies that cannot be used among healthy participants with normal cognitive development.

## Conclusion

This study has demonstrated that children with endocrinologic diseases exhibit lower performance in language, cognition, and mathematical skills compared to their peers with typical development. Specifically, children with endocrinological disorders scored lower on the TOLDP-4:T, indicating deficiencies in listening, organizing, speaking, grammar, and semantics. They also showed lower TNRT scores, reflecting limited phonological working memory, and lower WISC-R scores, indicating reduced cognitive function and inadequate mathematical performance as shown by the PST scores. These findings align with the existing literature that suggests that chronic diseases in childhood can negatively impact academic skills and cognitive development. Additionally, this study revealed that children with endocrinologic diseases were less effective in developing and implementing strategies necessary for solving complex problems. These findings suggest that endocrinologic disorders in childhood lead to declines in language, cognition, and mathematical skills negatively affecting academic performance.

## Limitations and perspectives

The limitations of this study include the presence of diverse types and etiologies of endocrinologic diseases among the participants, the insufficient number of participants, and the validity and reliability studies of the tool used for mathematical assessment being conducted for 15-year-old children. The study should be repeated with a larger group of participants. There is a need to develop standardized tools to examine the mathematical skills of Turkish school-age children. Children with endocrinologic diseases should be evaluated and monitored by a speech and language therapist for language, cognition, and mathematical problems alongside medical treatment.

Future studies should use larger and more homogeneous sample groups to examine the effects of childhood endocrinologic diseases on language, cognition, and mathematical skills in greater detail. Longitudinal studies that include different age groups and types of diseases can aid in better understanding the developmental processes of these children. Additionally, developing valid and reliable test tools specific to Turkish children to assess their language and mathematical skills is crucial. Controlled studies should be conducted to evaluate the effectiveness of intervention programs, and their impact on language, cognition, and mathematical skills should be investigated. Finally, the psychosocial interventions supporting the social and emotional development of children with endocrinologic diseases should be examined. A multidisciplinary approach should be adopted to improve the academic and social skills of these children.

## Data Availability

The datasets presented in this article are not readily available because the consent document for the data states that the data will not be shared with third parties. Requests to access the datasets should be directed to merve.savas@atlas.edu.tr.
